# Comprehensive analysis of genetic factors predicting overall survival in Myelodysplastic syndromes

**DOI:** 10.1038/s41598-022-09864-9

**Published:** 2022-04-08

**Authors:** Nehakumari Maurya, Purvi Mohanty, Somprakash Dhangar, Purvi Panchal, Farah Jijina, S. Leo Prince Mathan, Chandrakala Shanmukhaiah, Manisha Madkaikar, Babu Rao Vundinti

**Affiliations:** 1 Department of Cytogenetics, ICMR—National Institute of Immunohematology, K.E.M. Hospital Campus, Mumbai, Maharashtra 400012 India; 2 Department of Clinical Hematology, King Edward Memorial Hospital, Mumbai, Maharashtra India

**Keywords:** Cancer, Oncology

## Abstract

Myelodysplastic syndromes (MDS) are a group of clonal hematological disease with high risk of progression to AML. Accurate risk stratification is of importance for the proper management of MDS. Genetic lesions (Cytogenetic and Molecular mutations) are known to help in prognosticating the MDS patients. We have studied 152 MDS patients using cytogenetics and next generation sequencing (NGS). These patients were evaluated and as per cytogenetic prognostic group, majority (92.1%) of the patients classified as good (81.6%) and intermediate (10.5%) group. The NGS identified 38 different gene mutations in our cohort. Among 111 MDS patients with mutations, the most frequent mutated genes were *SF3B1* (25.2%), *SRSF2* (19%) *U2AF1* (14.4%) *ASXL1* (9.9%) *RUNX1* (9.9%) *TET2* (9%), *TP53* (9%), *ATM* (6.3%), *NRAS* (5.4%) and *JAK2/3* (5.4%). The survival analysis revealed that the mutations in *TP53*, *JAK2/3*, *KRAS*, *NRAS* and *ASXL1* were significantly (*P* < 0.05) associated with poor survival of the patients. The univariate cox and multivariate cox analysis of our study suggested that the age, marrow morphology, cytogenetic and gene mutations with IPSS-R should be considered for prognosticating the MDS patients. We have proposed M-IPSS-R which changed the risk stratification i.e. 66.3% patients had decreased risk whereas 33.75% showed increased risk compared to IPSS-R. The survival analysis also showed that the M-IPSS-R were more significant in separating the patients as per their risk than the IPSS-R alone. The change in risk stratification could help in proper strategy for the treatment planning.

## Introduction

Myelodysplastic syndromes (MDS) are a clonal hematopoietic stem cell disorder manifesting significant clinical heterogeneity and genetic diversity. MDS mainly characterized by varying degree of cytopenias due to ineffective hematopoiesis and dysplasia in one or more hematopoietic stem cell lineages. It is primarily a disease of the elderly with risk of transformation to acute myeloid leukemia (AML)^1,2^.

Chromosomal abnormalities, somatic mutations, and epigenetic changes represent key pathogenic defects in MDS. Recurrent chromosomal aberrations associated with distinct clinical outcomes, continue to remain the most important prognostic factors for treatment planning in MDS. However, approximately 50% of MDS patients are cytogenetically normal suggesting the presence of distinct molecular events that contribute to disease phenotype and transformation^3,4^.

Although, clinical risk stratification tools have been successful in prognostication of MDS patients, neither the International Prognostic Scoring System (IPSS) nor its revision (IPSS-R), consider somatic mutations which could not only aid in diagnosis of early-stage disease with ambiguous morphology but also account for clinical heterogeneity associated with inter-patient variability of harbored somatic mutations^5–7^. Comprehensive molecular studies have identified a set of recurrently mutated genes, associated with cellular processes, such as signal transduction, RNA splicing, epigenetic and transcriptional regulation. The current treatment protocols rely on IPSS-R for prognostication of MDS patients, however, there is a need for optimized treatment strategies for proper prognostic evaluation of individual patients owing to the clinical and biological heterogeneity associated with the disease.

Several studies have evaluated the prognostic significance of genetic lesions in MDS patients with varied findings in different study populations^1,2,8–10^. In last few decades the rapid development of high throughput technology like next generation sequencing has tremendously improved the genetic profiling of MDS^1,2,8–10^. The mutations identified majorly the genes involved in DNA methylation (*DNMT3A, TET2, IDH1/IDH2*), chromatin modification (*EZH2, ASXL1*), transcription factors (*RUNX1, GATA1/GATA2*), RNA splicing (*SF3B1, U2AF1, SRSF2* and *ZRSR2*) and signal transduction (*JAK2, KRAS/NRAS, CBL*)^8–14^. These recurrent mutations often occur in combination with distinct prognostic implications and are associated with leukemia transformation, response to hypomethylating agents and overall survival of the patients^8,11–14^. The cytogenetic profile of MDS has been explored in Indian subcontinent, however, the data on mutational profile of MDS is lacking^4,15,16^. The evolving data on mutations implicated in pathogenesis of MDS is continuously increasing^17–19^ and it also implies that targeted sequencing could offer cost-effective and treatment strategies for MDS patients. The incorporation of mutations in current prognostic scoring systems along with cytogenetic and clinical data is required and should be explored in different populations to define an inclusive scoring system for proper prognostic evaluation. The present study describes the mutational spectrum of MDS in India and its importance in disease prognostication.

## Results

### Patients characteristics

The clinical characteristics of MDS patient’s cohort of our study in comparison with Haferlach et al.^8^ summarized in Supplementary Table S1. The median age of our MDS group is lower (55 years vs. 72.8 years) and also a significantly (*p* < 0.001) high frequency of MDS were below 59 years (56.6% vs. 13.5%). A significantly (*p* < 0.001) high frequency (62%) of low risk MDS patients in our cohort compared to the Haferlach et al. study (54%).

In our study patients with IPSS-R score of ≤ 3.5 points^20^ were defined as lower risk MDS and patients with > 3.5 points as higher risk MDS.A high frequency [53.3% (81/152)] of higher risk patients were observed in our study compared to lower risk 71 (46.7%) patients. Among 86 younger (≤ 59 years) patients, a high frequency [57% (49/86)] of patients were of higher risk MDS. However, no significant difference was observed between the age and risk groups of MDS patients.

### Cytogenetic classifications of MDS patients

The chromosomal abnormalities were identified in 61 (40%) MDS in our study whereas Haferlach et al. reported 31.4% (Supplementary Table S1). Among chromosomally abnormal (N = 61) MDS patients, del(5q) [14 (23%)] was more frequent followed by del(20q) [13 (21.3%)]. The complex karyotypes were identified in 3 (5%) cases. Hence in our study majority of MDS patients were of Good cytogenetic risk [124 (81.6%)] as per IPSS-R cytogenetic risk classifications. More than one cytogenetic aberration was identified in 15 (24.5%) MDS patients (Supplementary Table S2).

### Gene mutations in MDS patients

Next generation sequencing revealed at least one non-synonymous gene mutation in 111 (73%) patients. In the current study different genomic lesions (203) were detected, which caused change in amino acid of coding regions. Among these mutations, 159 (77.9%) were single nucleotide substitution followed by deletions 21 (10.2%), insertions 12 (6.1%) and duplications 10 (5%) (Supplementary Table S6).

Among 152 MDS patients the median number of gene mutations was calculated as 1 and number of mutations identified in patients ranges from 0 to 6. The average gene mutations per MDS subtype was 1.02 (MDS-SLD, 46/45), 1.28 (MDS-MLD, 64/50), and 2.02 (MDS-EB, 93/46) (Supplementary Table S6). A high proportion [43.4% (66/152)] of patients from each MDS subtype harbored one gene mutation followed by 29.6% (45/152) patients had ≥ 2 mutations. High frequency [15% (23/152)] of patients having ≥ 2 mutation were of MDS with excess blast group (*p* = 0.014). Among cytogenetic group, a high frequency (76.5%) of gene mutations were identified in poor (7/8) and good risk (94/124) groups whereas intermediate (8/16) and very poor (2/4) risk group had 50% frequency of gene mutations (*p* = 0.081) (Table 1). Mutational distribution among age group of MDS patients revealed significantly (*p* = 0.038) a high frequency (83.1%, 49/59) of gene mutation in older MDS (> 59 years) patients than the younger (< 59 years) MDS patients (66.7%, 62/93) (Table 1). Correlation of risk group of MDS and gene mutations distribution revealed, high risk (IPSS-R score > 3.5) MDS patients had significantly (*p* = 0.002) higher incidence of gene mutations [84% (68/81)] than the low risk [60.6% (43/71)] patients (IPSS-R score ≤ 3.5).

**Table 1:** Correlation of cytogenetic risk group, IPSS R risk group and age group with incidence of mutations in MDS patients.

Groups (N = 152)	Mutational status in MDS risk subgroups		
	With mutations N (%)	Without mutations N (%)	*p* value
**Cytogenetic risk**			
Good (124)	94 (75.8)	30 (24.2)	0.081
Intermediate (16)	8 (50)	8 (50)	
Poor (8)	7(87.5)	1 (12.5)	
Very Poor (4)	2 (50)	2 (50)	
**IPSS-R risk group**			
Low (41)	24 (58.6)	17 (41.4)	> 0.05
Intermediate (68)	51 (75)	17 (25)	
High (31)	24 (77.5)	7 (22.5)	
Very high (12)	12 (100)	0	
**IPSS R score risk**			
≤ 3.5 Score (71)	43 (60.6)	28 (39.4)	0.002
> 3.5 Score (81)	68 (84)	13 (16)	
**Age**			
≤ 59 Years (93)	62 (66.7)	31 (33.3)	0.038
> 59 Years (59)	49 (83)	10 (17)	

Among the 111 patients with non-synonymous mutations, the most frequent mutated genes were *SF3B1* (25.2%), *SRSF2* (19%) *U2AF1* (14.4%) *ASXL1* (9.9%) *RUNX1* (9.9%) *TET2* (9%), *TP53* (9%), *ATM* (6.3%), *NRAS* (5.4%) and *JAK2/3* (5.4%) (Supplementary Fig. S1). Although distribution of gene mutations among age group revealed that older MDS patients had high incidence of *SF3B1* (28.6% vs. 21.8%), *U2AF1* (19.6% vs. 9.1%), *ASXL1* (12.5% vs. 7.3%), *TET2* (14.3% vs. 3.6%) and *DNMT3A* (7.1% vs. 1.8%) mutations, no statistical difference was observed when compared with the younger patients (*p* > 0.05) (Table 2). Distribution of gene mutations among risk group of MDS showed significant (*p* = 0.008) association of *TP53* gene mutation with high risk IPSS-R group (IPSS R score > 3.5) whereas *SF3B1* was associated with low risk (IPSS R score ≤ 3.5) MDS patients (*p* = 0.021). Though, the gene mutation frequency of *SRSF2* (23.5% vs. 14%), *U2AF1* (16.2% vs. 11.6%), *ASXL1* (13.2% vs. 4.7%), *RUNX1* (13.2% vs. 4.7%), *ATM* (7.4% vs. 4.7%) and *NRAS* (5.9% vs. 4.7%) was more in high risk MDS patients, there was no significant difference was observed between high and low risk groups (*p* > 0.05) (Table 3).

**Table 2:** Age wise distribution of gene mutations frequency in 111 MDS patients.

Genes	Frequency (N = 111)	Younger (N = 55) (≤ 59 years)	Older (N = 56) (> 59 years)	*p* value
SF3B1	28 (25.2%)	12 (21.8%)	16 (28.6%)	0.050
SRSF2	22 (19.8%)	14 (25.5%)	8 (14.3%)	0.176
U2AF1	16 (14.4%)	5 (9.1%)	11 (19.6%)	0.569
ASXL1	11 (9.9%)	4 (7.3%)	7 (12.5%)	0.357
RUNX1	11 (9.9%)	5 (9.1%)	6 (10.7%)	0.569
TET2	10 (9%)	2 (3.6%)	8 (14.3%)	0.311
TP53	10 (9%)	7 (12.7%)	3 (5.4%)	0.662
ATM	7 (6.3%)	5 (9.1%)	2 (3.6%)	0.990
NRAS	6 (5.4%)	3 (5.5%)	3 (5.5%)	0.150
JAK2/3	6 (5.4%)	5 (9.1%)	1 (1.8%)	0.990
DNMT3A	5 (4.5%)	1 (1.8%)	4 (7.1%)	0.311
KMT2D	5 (4.5%)	2 (3.6%)	3 (5.4%)	0.319
NOTCH1	5 (4.5%)	3 (5.5%)	2 (3.6%)	0.319
ZRSR2	5 (4.5%)	2 (3.6%)	3 (5.4%)	0.175
SETBP1	5 (4.5%)	2 (3.6)	3 (5.4%)	0.775
KRAS	4 (3.6%)	3 (5.5%)	1 (1.8%)	0.982
NPM1	4 (3.6%)	2 (3.6%)	2 (3.6%)	0.3
IDH1/2	3 (2.7%)	1 (1.8%)	2 (93.6%)	0.632
BCOR	3 (2.7%)	1 (1.8%)	2 (3.6%)	0.14
Other genes	2–1 (1.8–0.9%)	–	–	–

**Table 3:** Distribution of gene mutations in high and low risk of 111 MDS patients.

Genes	Frequency (N = 111)	Low risk (N = 43) IPSS Score ≤ 3.5	High risk (N = 68) IPSS Score > 3.5	*p* value
SF3B1*	28 (25.2%)	16 (37.2%)	12 (17.6%)	0.026
SRSF2	22 (19.8%)	6 (14%)	16 (23.5%)	0.328
U2AF1	16 (14.4%)	5 (11.6%)	11 (16.2%)	0.588
ASXL1	11 (9.9%)	2 (4.7%)	9 (13.2%)	0.198
RUNX1	11 (9.9%)	2 (4.7%)	9 (13.2%)	0.198
TET2	10 (9%)	5 (11.6%)	5 (7.4%)	0.506
TP53**	10 (9%)	0	10 (14.7%)	0.006
ATM	7 (6.3%)	2 (4.7%)	5 (7.4%)	0.706
NRAS	6 (5.4%)	2 (4.7%)	4 (5.9%)	1.0
JAK2/3	6 (5.4%)	4 (9.3%)	2 (2.9%)	0.206
DNMT3A	5 (4.5%)	1 (2.3%)	4 (5.9%)	0.647
KMT2D	5 (4.5%)	2 (4.7%)	2 (2.9%)	1.0
NOTCH1	5 (4.5%)	3 (7%)	2 (2.9%)	0.374
ZRSR2	5 (4.5%)	1 (2.3%)	4 (5.9%)	0.647
SETBP1	5 (4.5%)	0	5 (7.4%)	0.154
KRAS	4 (3.6%)	1 (2.3%)	3 (4.4%)	1.0
NPM1	4 (3.6%)	2 (4.7%)	2 (2.9%)	0.640
IDH1/2	3 (2.7%)	2 (4.7%)	1 (1.5%)	0.558
BCOR	3 (2.7%)	0	3 (4.4%)	0.282
Other genes	2–1 (1.8–0.9%)	–	–	–

Among the different functional groups in 111 MDS patients, the older patients had a significantly (23.2% vs. 7.3%, *p* = 0.033) higher incidence of DNA methylation- and hydroxyl-methylation related gene (*DNMT3A, IDH1/2* and *TET2*) mutations while younger MDS patients had a higher (27.3% vs. 16.1%, *p* = 0.173) incidence of activated signaling gene (*CBL, GNAS,JAK2/3, KRAS, NRAS, PTPN11* and *NOTCH1*) mutations and transcriptional factor related genes (*CUX1, GATA2, IKZF1, RUNX1, PHF6, ETV6, TP53* and *WT1*) (27.3% vs. 17.9%, *p* = 0.263), however, statistically significant difference was not found between both the groups. Although there was a trend of higher incidence of histone modifying genes (*ASXL1, BCOR, EZH2, AND KMT2D*) (21.4% vs. 14.5%, *p* = 0.46) and RNA spliceosome gene (*SF3B1, SRSF2, U2AF1* and *ZRSR2*) (64.3% vs. 60.0%, *p* = 0.698) mutations in older patients, no significant difference was observed when compared with the younger patients (Supplementary Fig. S2).

The *NPM1* gene mutations were identified in (3.6%, 4/111) MDS patients. The patient’s characteristics are summarized in (Supplementary Table S3). These patients were classified as per IPSS-R criteria, and 2/4 (50%) were classified as high risk, 1 (25%) as low risk, and 1 (25%) as intermediate risk. According to WHO 2016 classification 2 (50%) were classified as MDS with multi-lineage dysplasia and 2 (50%) as MDS with excess blast (1 = MDS-EB-1 and 1 = MDS-EB-2) The median BM blast percentage was 5.5% (range 2–19%) and 4 patients had a normal karyotype. All *NPM1* mutated patients in our study are females (100% vs. 43.9%, *p* = 0.04), *NPM1*-mutated MDS patients were younger (median age, 48 vs. 56 years, *p* < 0.67), had median BM blast percentage at diagnosis (5.5% vs. 3%, *p* < 0.928), and had a 100% frequency of normal karyotype (100% vs. 58.8%, *p* < 0.149) compared with *NPM1* wild-type patients. The median age, marrow blast percent and frequency of normal karyotype were not statistically significant different than *NPM1* wild type patients. It was observed that out of 4 *NPM1* mutation patients, three patients had other gene mutations like *IDH2, BCOR with U2AF1* and *NRAS with IDH2* and all three had poor prognosis, whereas patient with one *NPM1* mutation showed good response to intensive chemotherapy.

### Overall survival of MDS patients

**Figure 1:**
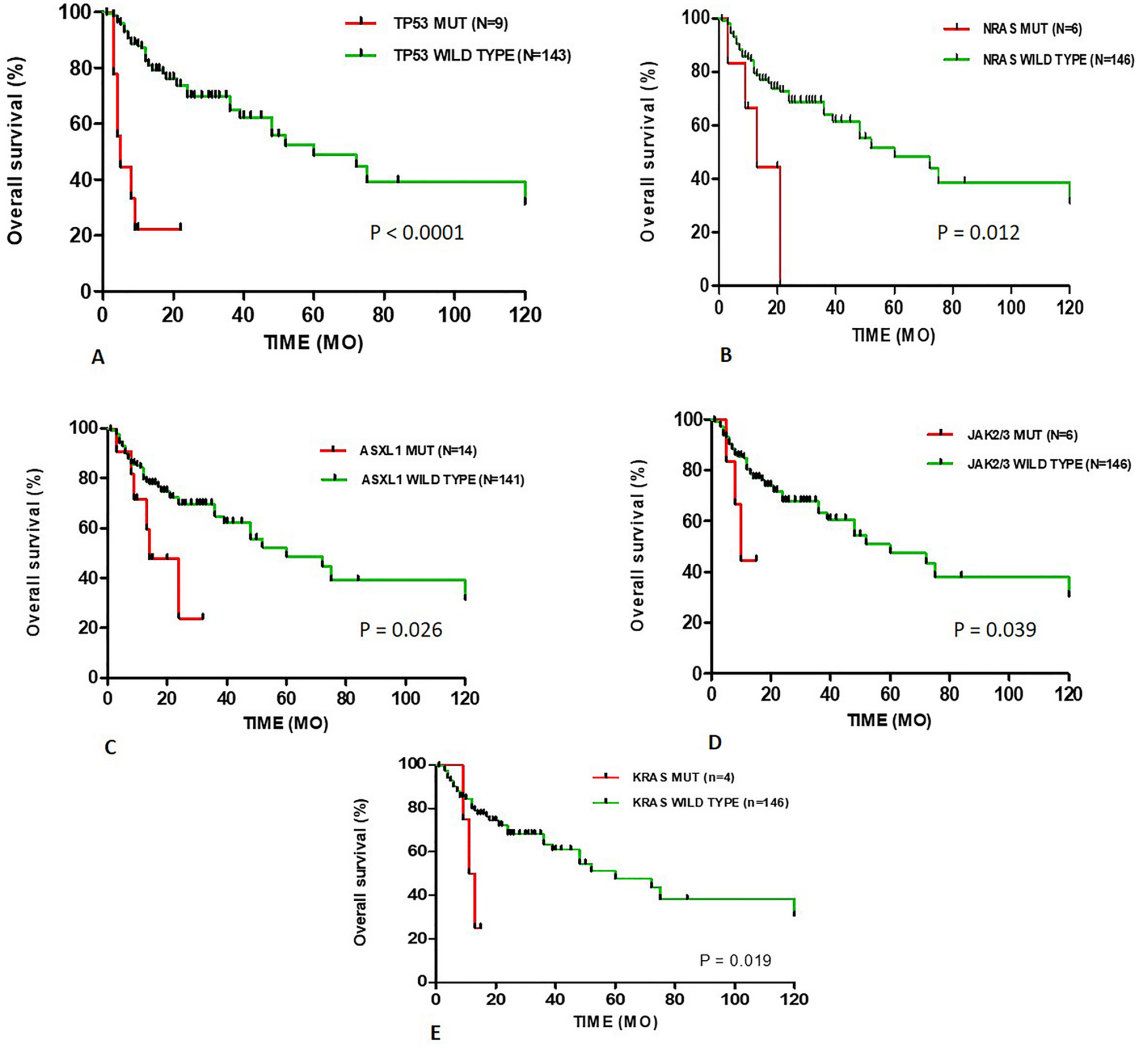
Kaplan–Meier curves of overall survival (OS) of patients with (**A**) TP53 mutations, (**B**) NRAS mutations, (**C**) ASXL1 mutations, (**D**) JAK2/3 mutations and (**E**) KRAS mutations had worse OS than wild type patients.

The gene mutation status of patients associated with their overall survival was determined in 152 MDS patients using Kaplan–Meier (K–M) survival curve with Log Rank analysis. We considered median survival < 25 months as a poor survival in our study. Among 152 MDS patients, 53 (35%) died, of which 40/53 (75.5%) had at least one non synonymous gene mutation, whereas 13/53 (24.5%) had no gene mutations (*p* = 0.713). The survival analysis revealed, mutations in *TP53* (OS = 5 month, *p* =  < 0.0001), *JAK2/3* (OS = 10 months, *p* = 0.039), *KRAS* (OS = 12 months, *p* = 0.019), *NRAS* (OS = 13 months, *p* = 0.012) and *ASXL1* (OS = 14 months, *p* = 0.026) were significantly associated with poor survival of the patients (Table 4; Fig. 1). The gene variants in *NOTCH1* (OS = 13 months, *p* = 0.11), *NPM1* (OS = 21 months, *p* = 0.41) and *RUNX1* (OS = 24 months, *p* = 0.075) also presented with poor survival of patients. There was no statistically significant difference in OS among the patients with *NOTCH1, NPM1* and *RUNX1* gene mutations and wild type MDS patients.

**Table 4:** Kaplan–Meier Survival analysis of patients with mutations in MDS.

Mutation variable	Mutational status	N	Median OS (Months)	*p* value
TET2	MT	10	31.5	0.136
	WT	142	60	
DNMT3A	MT	5	NR	0.79
	WT	147	60	
ASXL1	MT	11	14	0.0261
	WT	141	60	
RUNX1	MT	11	24	0.075
	WT	141	72	
TP53	MT	9	5	< 0.0001
	WT	143	60	
SF3B1	MT	28	48	0.79
	WT	124	72	
SRSF2	MT	22	52	0.121
	WT	130	48	
U2AF1	MT	16	NR	0.402
	WT	136	52	
JAK2/3	MT	6	10	0.039
	WT	146	60	
KRAS	MT	4	12	0.019
	WT	148	60	
NRAS	MT	6	13	0.012
	WT	146	60	
NOTCH1	MT	5	13	0.11
	WT	147	60	
NPM1	MT	4	21	0.41
	WT	148	60

### Predictors of OS

The cox proportional hazards regression analysis (univariate and multivariate) was performed to identify independent predictors of OS. Factors considered for analysis was age (≤ 60 vs. > 60), marrow dysplasia (SLD vs. MLD vs. EB), cytogenetic category (good vs. intermediate vs. poor/very poor), blast percent (5% vs. > 5%), IPSS-R (low vs. high risk group), and gene mutations including *TET2, ASXL1, TP53, NRAS, KRAS, RUNX1* and *NOTCH1*.The univariate analysis demonstrated that, the age (HR = 1.88, *p* = 0.022), marrow dysplasia (HR = 1.85, *p* = 0.001) cytogenetic group (HR = 1.67, *p* = 0.013) and IPSS R (HR = 2.0, *p* = 0.014) were significantly unfavorable prognostic factors for patients overall survival whereas blast percent showed hazard ratio more than 1 indicating increased risk but not (HR = 1.43, *p* = 0.2) statistically significant. Mutations in *ASXL1* (HR = 2.55, *p* = 0.034), *TP53* (HR = 6.29, *p* < 0.000), *RUNX1* (HR = 2.37, *p* = 0.035), *NRAS* (HR = 3.40, *p* = 0.02) and *KRAS* (HR = 3.69, *p* = 0.031) negatively affected OS of patients, while *NOTCH1* and *TET2* had hazard ratio of > 1 indicative of increased risk (*p* > 0.05) but not statistically significant (Table 5).

**Table 5:** Univariate and multivariate analysis of OS in MDS patients.

	Univariate			Multivariate*		
	Hazard ratio	95.0% CI for HR		Hazard ratio	95.0% CI for HR	
		Lower	Upper		Lower	Upper
AGE in years (≥ 60 vs. < 60)	1.885	1.096	3.241	1.718	0.917	3.220
Marrow morphology (SLD vs. MLD vs. EB)	1.852	1.284	2.671	2.814	1.237	6.399
Cytogenetic group (good vs. inter vs. poor)	1.675	1.113	2.52	1.953	1.2	3.179
BLAST % (< 5% vs. > 5%)	1.433	0.825	2.489	0.473	0.137	1.631
IPSS R (low risk vs. high risk)	2.001	1.150	3.483	0.526	0.229	1.211
TET2 Mutation	1.821	0.810	4.092	1.811	0.640	5.122
ASXL Mutation	2.556	1.075	6.075	0.604	0.197	1.856
TP53 Mutation	6.290	2.740	14.442	5.947	2.174	16.266
RUNX1 Mutation	2.376	1.065	5.304	2.879	1.164	7.122
NRAS Mutation	3.403	1.209	9.582	2.616	0.536	12.759
KRAS Mutation	3.697	1.129	12.106	1.105	0.189	6.465
NOTCH1 Mutation	2.507	.768	8.186	3.875	1.137	13.124

*Multiple variables were selected for the Cox proportional hazard model: age (≥ 60 vs. < 60 year), marrow morphology (SLD vs. MLD vs. EB), bone marrow blast, IPSS-R, and TET2, ASXL1, TP53, RUNX1, NRAS, KRAS and NOTCH1 gene mutations.

The multivariate analysis revealed that the cytogenetic (HR = 1.95 *p* = 0.007), marrow morphology (HR = 2.8, *p* = 0.014), *TP53* (HR = 5.9, *p* = 0.001), *RUNX1* (HR = 2.87, *p* = 0.022) and *NOTCH1* (HR = 3.8, *p* = 0.03) were significantly associated with poor OS (Table 5).

### Incorporation of risk factors into IPSS-R

**Figure 2:**
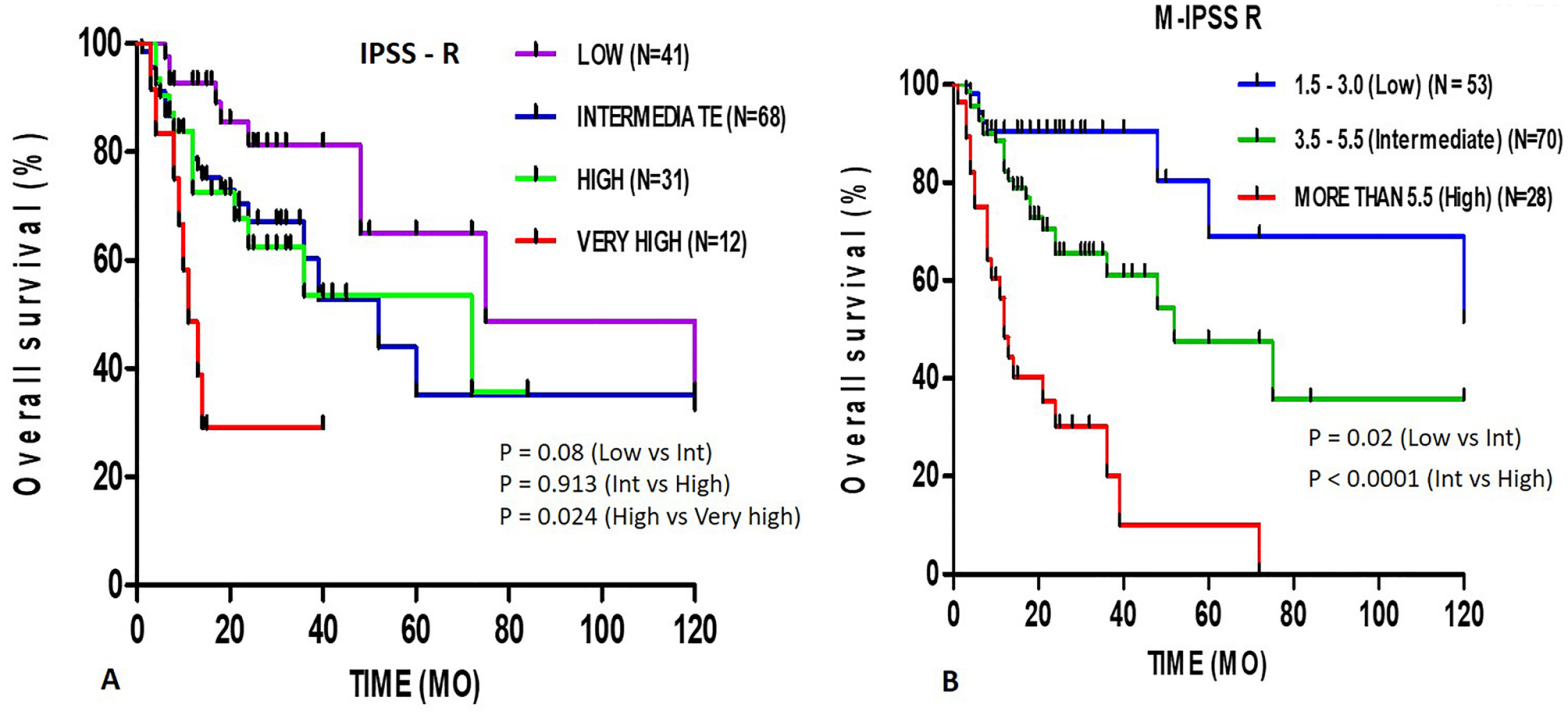
Kaplan–Meier curve of survival according to (**A**) IPSS-R risk group and (**B**) M-IPSS-R risk group system.

The IPSS-R information of 152 MDS patients was used for classification of patients into low risk, intermediate risk, high risk, and very high risk, with no significant difference in survival, especially between the low risk (OS = 75 months) versus intermediate risk (OS = 52 months) risk (*p* = 0.08) and intermediate risk versus high-risk (OS = 72 months) subgroups (*p* = 0.913) (Fig. 2A) The MDS patients with genetic lesions increased to 88.8% when gene mutations (73%) data was combined with cytogenetics (40%).

The cox proportional hazards regression analysis suggested that age, marrow morphology with number of lineage dysplasia and gene mutations are significant predictors for OS, we then included these factors into IPSS-R and proposed new scoring system, named (Molecular) M-IPSS-R system. Scoring for each proposed factors and selected gene mutations described in Table 6 and Supplementary Table S7. In our proposed model for single gene mutation the score was given as 1 and the score was increased with increase in number of mutations. Based on the M-IPSS-R model, we have re-classified the 152 MDS patients into Low Risk (score from 1.5 to 3), Intermediate Risk (score 3.5–5.5) and High risk (score > 5.5). The K-M survival analysis showed significant difference between OS of Low risk (OS = Not reached 50% median survival) versus Intermediate risk (OS = 52 months) (*p* = 0.02) and between intermediate risk versus high (OS = 12 months) (*p* < 0.0001) suggests that these risk classified patients were more accurately prognosticated into 3 different prognostic subgroups (Fig. 2B).

**Table 6:** M-IPSS-R proposed scoring system for prognosis of MDS patients.

Prognostic variable	0	0.5	1	2	3	4	5
Gene mutations^¶^			*	**	***	****	> ****
Marrow morphology		SLD	MLD	5–10% blast	> 10% blast		
Cytogenetics			Good	Intermediate	Poor	Very poor	
Age	≤ 30	31–50	51–60	> 60			

^¶^Selected genes TET2, ASXL1, TP53, RUNX1, NRAS, KRAS, NOTCH1.

*Presence of any 1 gene mutation from selected genes;

**Presence of any 2 gene mutation from selected genes;

***Presence of any 3 gene mutation from selected genes;

****Presence of any 4 gene mutation from selected genes.

>****Presence of any more than 4 gene mutation from selected genes.

## Discussion

MDS is a heterogeneous group of hematologic neoplasms which may occur de novo or secondary to various offense to the bone marrow. MDS is usually an elderly disease and the incidence increases with advancing age. In our study the median age of MDS patients at diagnosis was 55 years with range from 16 to 90 years, which is similar with the reports from Asian countries; India (55 years)^16^, Pakistan (51 years)^3^ and China (62, 51 years)^21,22^. On the other hand, median age at diagnosis in our study cohort is different from US (77 years)^23^, Europe (76 years)^24^ and France (~ 78 years)^24^. The younger patients (≥ 55 years), were predominant (55.9%) in our study, which is similar to China MDS populations (~ 52%)^22^, but different from USA (13.5%)^25^, Greece (28.1%)^26^ and Poland (~ 12)^27^. As per Surveillance Epidemiology and End Results (SEER)-Medicare database, also reported that the median age in Asian countries is earlier than that of Western countries^24^. The trend towards MDS development in younger age in India and Asian countries may be due to different genetic susceptibilities among ethnic groups, geographical, dietary reasons and importantly occupational and environmental stress like toxin exposures.

The MDS patients recruited in our study were classified as per the 2016 revision of the WHO classification of myeloid neoplasms and acute leukemia^28^. Our study cohort showed a high frequency (32.6%) of MDS-MLD subgroup (Supplementary Table S1) followed by MDS with excess blast (EB1 and EB2) (30.2%) among other MDS subgroups. Our results were similar with the study reported from the Pakistan (26.9%, 22%)^3,29^ and China (57.6%, 63.6%)^21,22^. The studies^3,21,22,29^ also reported that the MDS excess blast (EB1 and EB2) was the second most frequent subgroups (32.6%, 39%, 15.3%, and 30%). On the other hand reports from western countries (USA, Switzerland and Netherland)^24^ reported MDS-U, more frequent (61%, 60.7% and 42.6%) MDS subgroup and the MDS-EB frequency is found to be less (16.3, 17.5%, 20.2% vs. 32.6%, 39%, 15.3%, 30%) compared with the Asian countries^3,21,22,29^. The difference in frequency of MDS subgroups may be due to the different ethnicity, population of the study, and/or sensitivity of the methodology adopted for identification of MDS morphology.

Clonal chromosome aberrations play an important role in diagnosis and prognosis of malignancies. The frequency of chromosomal abnormalities in MDS patients are ranging from from 35 to 48%^16,30–33^. Our study detected chromosome aberrations in 40% (61/152) of the MDS patients, which is in agreement with the previous studies. In the present study most frequent isolated chromosomal abnormality was del(5q) (23%, 14/61) concurrent frequent (57.1%, 4/7) chromosome abnormality was reported by Pakistan study group^29^, however Rashid et al.^32^ from Pakistan and Xueying Wang et al.^22^ from China reported, trisomy 8 as the most common (23.3%, 25.2%) cytogenetic abnormality respectively. Chaubey et al.^16^ from India reported monosomy 7 as the most frequent cytogenetic abnormality detected in 31.6%, followed by del 5q in 21% and trisomy 8 in 16%. We also compared the cytogenetic data with the study reported by Papaemmanuilʼs et al. 2013^12^. The chromosome aberration frequency almost similar except del(20q) (3% vs. 8.5%) and complex karyotype (8% vs. 1.9%) when calculated among MDS cohort. This difference in frequency of chromosome aberrations may be due to younger age MDS cohort. Hence our study indicate that the type and frequency of aberrations varies with ethnic and regional differences. The prognosis of the patients were assessed as per IPSS-R scoring system which combines the scores of 5 main factors including hemoglobin, platelets, neutrophil count, cytogenetics and marrow blast, among these cytogenetics has the highest value^7^. Accordingly, intermediate risk patients (45%) (Supplementary Table S1) were predominant than high/very high risk (28.9%) and low risk (27%) patients in our study, whereas study from china reported a high frequency (38%) of low risk patients^7^. Jiang et al.^24^ did comparison study between Asian MDS and Western MDS with respect to their epidemiology, clinical, biological and genetic characteristics and concluded that, in Asian countries more patients are distributed in intermediate, high and very high risk group compared to Europe and North America. In our study we observed that, 75% of the intermediate risk patients had at least one gene mutation which was very similar to the frequency of high risk patients (77.5%) having gene mutations (Table 1). The IPSS-R scoring system was developed in patients without treatment. The insignificant difference (*p* = 0.913) (Fig. 2A) of OS between intermediate and high risk in our study may be due to patients treated with HMAs, chemotherapy or HSCT. However, the genetic data may give better understanding of OS.

The advancement in molecular biology technology has improved the genome-wide analysis of genetic mutations in MDS^8,34^. Gene mutations frequency differ among different countries from 51 to 91.4% in MDS patients^24^. In our study, we have identified mutations in 111 (73%) of MDS patients had at least one mutation. Overall mutation frequency among MDS cohort is similar (78%, 576/738) to the study reported by Papaemmanuilʼs data^12^, whereas other study group from China reported 91.4% mutations^35^ and Haferlach et al.^8^ reported 89.5%. The most frequently mutated genes among 111 patients were *SF3B1*, *SRSF2, U2AF1*, *ASXL1*, *RUNX1*, *TET2*, *TP53*, *ATM*, *NRAS* and *JAK2/3* accounting for more than 5% frequency in these patients (Supplementary Table S6). Though similar genes were mutated among different^8,12^ MDS cohort, in our study low frequency of TET2 (9% vs. 37.6% vs. 30.2%), ASXL1 (9.9% vs. 26.6% vs. 16.6), DNMT3A (4.5% vs. 15% vs. 12%) were observed. However a high frequency of U2AF1 (14% vs. 8.6% vs. 8%) compared to other studies (Supplementary Fig. S3). These differences in frequency could be explained by difference in MDS cohort characteristics such as median age (55, 72.8^8^, 68^12^ years), marrow morphology [(SLD—29.3, 16%^8^, 33%^12^), (MLD—32.6%, 40%^8^, 25%^12^), (Excess blast—30.2%, 40.6%^8^, 23%^12^)]. International working group for the prognosis of MDS, reported that approximately half of MDS patients carry somatic mutations in splice factor genes, and of these, *SF3B1* is the most commonly mutated gene^36^. Our data is concurrent with the previous study and showed that the out of 111 mutated patients 58.5% of patients had RNA splice factor gene mutations and of these *SF3B1* gene mutations were reported with high frequency (25.2%). The results showed that MDS patients with *SF3B1* mutations were predominant in the low-risk group. The patients with *NRAS, KRAS, IDH2, TP53, RUNX1, ASXL1, ZRSR2, U2AF1, ATM, SETBP1* and *SRSF2* mutations were predominant in the high-risk group (Table 3). Haferlach et al.^8^ and Makishima et al.^37^ also reported these mutations to be frequent across MDS high risk patients. In our study 6 patients with MDS-RS morphology *SF3B1* gene mutations were identified in 5 patients (83%) which is similar with the studies^8,36^. However SF3B1 mutations were also identified in 22 other MDS patients in our cohort. As these patients had low percentage of ring sideroblast (> 14%) and hence failed to classify as MDS-RS as per WHO classification. Xiong et al.^38^ suggested that the SF3B1 mutation, but not the presence of ring sideroblasts, identified a distinct subtype and showed independent prognostic value on survival and leukemia transformation.

The pathogenic role of recurrent gene mutations in MDS has been suggested by several groups^8,12,34–36^. These gene mutations are drivers for disease evolution, i.e. from asymptomatic clonal hematopoiesis to MDS, and, ultimately progression to AML. These genes have been classified by different study groups^39–41^, into a limited number of cellular processes, including RNA splice factor genes, epigenetic and transcriptional regulation, and signal transduction. Our study showed that the older patients had a significantly (*p* = 0.03) higher incidence of DNA methylation- and hydroxyl-methylation related gene (23.2%) (*DNMT3A, IDH1/2 and TET2*) mutations, while younger MDS patients had higher incidence of activated signaling genes (27.3%) (*CBL, GNAS, JAK2/3, KRAS, NRAS, PTPN11* and *NOTCH1*) mutations and transcriptional factors related genes (27.3%) (*CUX1, GATA2, IKZF1, RUNX1, PHF6, ETV6, TP53 and WT1*), however there was no significant difference was observed between both the age groups (Supplementary Fig. S2). Therefore, it is evident from our study that the detection of mutations can give useful genetic information that may be clinically applicable to current treatment methods.

In our study the *NPM1* gene mutations were identified in 4 (2.6%) female MDS patients (Supplementary Table S6) including MDS MLD (n = 2), MDS-EB (n = 2) and with normal karyotype (Supplementary Table S3). Guillermo Montalban-Bravo et al.^42^ studied a large cohort of MDS and reported lower frequency (n = 31/1900, 1.6%) of *NPM1* gene mutations, predominantly identified in females (55% vs. 33%, *p* = 0.02) and with high frequency of normal karyotypes (81% vs. 47%, *p* = 0.001). Though we have also identified lower frequency (4/152, 2.6%) of *NPM1* gene mutations, these patients need to be characterized carefully as these patients are prone to develop AML. Nucleophosmin 1 (*NPM1*) is a nucleolar protein involved in multiple cell function and protein–protein interactions^43^. The *NPM1* mutations are detected in 20–30% of AML also in 50–60% of karyotypically normal AML patients. Presence of *NPM1* mutations in AML is known to be associated with favorable outcomes when treated with intensive chemotherapy^44^. MDS and MDS/MPN patients have poor clinical course if presented with *NPM1* mutations with a high rate of AML transformation^45^. The distinction between AML and MDS is defined based on blast percent margin of 20% blasts and accordingly the treatment decisions changes. Hence, it is important to understand *NPM1* mutant MDS patients, as these patients are more likely to progress into AML compared with *NPM1* wild type MDS patients, regardless of blasts percentage. Study groups have suggested that *NPM1* mutations in myeloid neoplasms may classify as AML, even in the presence of, < 20% bone marrow (BM) blasts^45^.

In our study we considered, patient’s median OS below 25 months as a poor survival of MDS patients. Kaplan–Meier survival analysis revealed *ASXL1, TP53, KRAS, NRAS* and *JAK2/3* mutations were significantly (*p* < 0.05) associated with poor survival and prognosis independent of IPSS, similar result was reported by Jiang et al.^46^ and Bejar et al.^47^ study group. In our study we have observed that *KRAS* and *NRAS* mutations were frequent in high risk patients, Muhammad et al.^48^ showed, MDS patient with RAS gene mutation progressed to AML and its unfavorable indicator of survival in AML. Gene mutations in *RUNX1* and *NOTCH1* also showed poor survival but no statistical (*p* > 0.05) difference among wild type patients (Table 4). The *RUNX1* mutation was predominant (13.2%) versus (4.7%) (Table 3) in high risk patients, of our study and same frequency has been reported by Bejar et al.^47^ and also showed shorter survival. The *NOTCH1* is known to be associated with leukemogenesis in lymphocytic leukemias and has been reported more frequent in AML than MDS and showed poor patient’s survival^49^. In case of *TET2* mutation our study fail to show the association of *TET2* mutation with patient’s survival may be due to low number (N = 10) of patients or higher response to HMA^14^. Our result was similar to study from Jiang et al. who suggested that *TET2* mutation burden has association with patient’s OS and not the *TET2* mutation status^50^. Overall, the patients with mutational factors had shorter OS in comparison with those without such mutational factors within the same IPSS-R risk group. The genetic mutation information of patients could help to identify low risk MDS with poor survival. Also, the patient’s prognosis was deteriorating with increase in number of mutations. Hence identification of mutations in low risk is important for management of the disease.

Several study groups^1,2,8^ have suggested inclusion of gene mutation in IPSS-R prognosis system. Haferlach et al.^8^ considered the predictors such as age, gender, IPSS-R and 14 different gene mutations, built a novel prognostic model (model-1) and separating patients into four risk groups which showed significantly different 3-year survival rate. Nazha et al.^51^ incorporated *EZH2, SF3B1,* and *TP53* mutations with IPSS-R and improved the predictive ability in MDS. We also proposed M-IPSS R scoring system (Table 6) which included predictors like gene mutations (*TET2, ASXL1, TP53, RUNX1, NRAS, KRAS,* and *NOTCH1*), age, number of lineage dysplasia and cytogenetic risk classification as univariate and multivariate cox regression analysis demonstrated that these factors are associated with poorer OS and increased hazard risk. Genes like *EZH2, BCOR, PTPN11* and *CBL* were not consider for prognosis predictors as low frequency (1, 0.9%), (3, 2.7%), (2, 1.8%) and (2, 1.8%) was observed in cohort respectively. The 152 MDS patients of our study were re-classified as per M-IPSS-R scoring into low risk, intermediate risk and high risk. Log rank analysis showed significant difference between their survival curve and median OS of intermediate risk patients was 52 months and 12 months of high risk patients, while low risk patients didn’t reach the 50% median survival (Fig. 2B). The significance of proposed M-IPSS R scoring system was highlighted by comparing the change in risk stratification of patients with their respective IPSS R risk. 66.3% (53/80) patients had decreased risk and 33.75% (27/80) patients had increased risk (Supplementary Table S4).

In summary, *ASXL1, TP53, RUNX1, NRAS, KRAS, NOTCH1* and *TET2* mutations along with number of lineage dysplasia are important predictors for survival of MDS. Our study highlights that integrating mutation status, lineage dysplasia, age into IPSS R may improve risk stratification of patients with MDS and assist in identification of those with worse than expected prognosis for more aggressive treatment.

## Materials and methods

### Subjects

One fifty-two (152) primary MDS patients including 83 males and 69 females referred to our laboratory from various centers in India were enrolled in the study. Patients with, secondary/therapy-related MDS, toxic bone marrow damage, and congenital bone marrow failure syndromes were excluded from the study. The clinical and demographic details were recorded from patient’s medical records. Bone marrow aspirate (BMA) and peripheral blood (PB) samples were collected in heparin (4 cc) and EDTA (4 cc) vacutainers. Informed written consent was obtained from the study participants. The protocols of the study were approved by Institutional Ethics Committee on human subjects of ICMR-National Institute of Immunohaematology, Parel, Mumbai, India and all the methods were performed in accordance with the relevant guidelines and regulations.

### Bone marrow morphology

Giemsa Staining of BMA/PB Smears was carried out as per standard procedure to classify MDS patients according to clinico-pathomorphological criteria of WHO Classification (2016)^28^. The type and degree of dysplasia in myeloid lineages was also evaluated by experienced hematologists for IPSS based prognostication. The dysplastic features (pseudo-pelger neutrophils, ring sideroblasts, micromegakaryocytes and increased blast count) were assessed in minimum 10% of the nucleated cells in the lineage for significant dysplasia. At least 500 and 200 cells were evaluated from marrow and blood respectively.

### Conventional cytogenetic study

The BMA (2 ml) samples were cultured for 24–72 h in F-10 nutrient media (Sigma-Aldrich, USA) with 20% fetal bovine serum (FBS). The bone marrow aspirate were also directly harvested after arresting with colchicine (Sigma-Aldrich, USA) (50 μg/ml). The cultures were fixed with methanol: acetic acid (3:1v/v) after treating with 0.075 M hypotonic solution (KCl). The chromosomal preparations were obtained by dropping on pre chilled slides followed by aging of the chromosomes for 48 h at room temperature then subjected to GTG banding. The chromosomal analysis was carried out from minimum 20 metaphases from each case and karyotyped according to International System for Human Cytogenomic Nomenclature (ISCN) 2020.

### Fluorescence in-situ hybridization (FISH)

FISH was carried out using standard protocol, briefly the cells/metaphases and FISH probe were denatured at 80 °C, hybridized at 37 °C overnight. The FISH probes; (Vysis Inc, Downers Grove, IL, USA): (1) LSI EGR1 Spectrum Orange (SO)/D5S23, D5S721 Spectrum Green (SG) dual color probe for chromosome 5; (2) LSI D7S486 (7q31) SO/CEP 7 SG probes for chromosome 7; (3) CEP 8 (D8Z2) SO/for chromosomes 8 and (4) LSI D20S108 (20q12) SO probe for chromosome 20 were used in the study. The excess non-hybridized probes were washed out with wash solutions kept at 80 °C followed by nuclear counterstaining with DAPI for 15–20 min at room temperature. Analysis was carried out under fluorescence microscope (Nikon 90i) and digital images were analysed using GenASIs applied spectral imaging systems software (Applied Spectral Imaging, Israel). A total of two hundred intact, non- overlapping nuclei were assessed by 2 independent investigators and the percentages of positive nuclei were averaged.

### WHO classification of MDS patients and prognostic risk stratification

The patients were diagnosed and sub-grouped according to WHO 2016 classification criteria by well-trained haematologists. The risk stratification of the patients were carried out according to IPSS R scoring system^7^ considering the cytogenetic category, bone marrow blast percentage and complete blood count of patients. The treatment details and follow-up data of MDS patients for, survival analysis was collected and recorded during the disease course.

### Next generation sequencing

The genomic DNA was extracted from BMA and/or PB collected in EDTA tube, using QIAmp DNA blood mini kit (Qiagen). The custom capture kit was used for selective target enrichment followed by clinical exome sequencing at the Med-Genome Labs Pvt Ltd, Banglore, India. The libraries were prepared and sequenced at mean depth of 200–250X on Illumina sequencing platform with a gene coverage of approximately 98% to 100%. After sequencing, the sequences were obtained and aligned using BWA program^52,53^ to human reference genome (GRCh37/hg19) followed by analysis using Picard and GATK version 3.6^54,55^ to identify clinically relevant variants. VEP program^56^ was used for gene annotation of the variants against the Ensembl release 91 human gene model^57^.

### In-silico analysis

The prediction tools such as PolyPhen-2 (http://genetics.bwh.harvard.edu/pph2/), SIFT (https://sift.bii.a-star.edu.sg/), Mutation Taster2 (https://www.mutationtaster.org/), Mutation Assessor (http://mutationassessor.org/r3/) and Likelihood Ratio Test (LRT) was used to calculate the variants effect. Non-synonymous variants with damaging effect were considered in our study. The list of 246 genes covered in the panel is given in the Supplementary Table S5.

### Follow up of the patients

Follow up study was carried out on patients undergoing MDS specific treatment (different for low-risk and high risk MDS groups) for the period of the study. The response to treatment was assessed as per International Working Group (IWG) standardized response criteria^58^ for evaluating clinically significant responses in MDS.

### Statistical analysis

The descriptive statistics was used to summarize demographic and characteristics of MDS patients. Fisher’s exact test or Pearson’s χ^2^ test was used for the analysis of frequencies. The time of diagnosis to death due to any cause since last follow-up was considered as overall survival (OS). During the last follow up data were censored. Impact of genetic lesions on overall survival was performed by Kaplan–Meier (K–M) survival curve with Log Rank analysis using GraphPad Prism 5. Cox proportional hazard model was used for univariate and multivariate analysis using SPSS version 20 software (IBM Corp., Armonk, NY, USA) from the Survival package. The variates selected were age (≤ 60 vs. > 60), marrow dysplasia (SLD vs. MLD vs. EB), cytogenetic category (Good vs. intermediate vs. poor/very poor), blast percent (5% vs. > 5%), IPSS-R (low vs. high risk group), and gene mutations including *TET2, ASXL1, TP53, NRAS, KRAS, RUNX1* and *NOTCH1*. The predictors from the cox model analysis and with reference to Haferlach et al., was used to construct M-IPSS-R model. Patients risk were reclassified as low Risk (score from 1.5 to 3), Intermediate Risk (score 3.5–5.5) and High risk (score > 5.5) as per this model. This model was compared with IPSS-R with K–M survival study. The *p* < 0.05 was considered as statistically significant.

### Ethics approval and consent to participate

The written informed consent was taken from all the patients by the Declaration of Helsinki before enrollment in the study.

### Consent for publication

All the authors jointly prepared the manuscript and approved for the publication.

## Supplementary Information

Supplementary Information 1.

Supplementary Information 2.

Supplementary Information 3.

Supplementary Information 4.

Supplementary Information 5.

Supplementary Information 6.

Supplementary Information 7.

Supplementary Information 8.

Supplementary Information 9.

Supplementary Information 10.
