# Supplementary figures and images for: Comprehensive analysis of genetic factors predicting overall survival in Myelodysplastic syndromes
Source: Sci Rep. 2022 Apr 8;12:5925. doi: 10.1038/s41598-022-09864-9 (PMC8993876; doi:10.1038/s41598-022-09864-9)

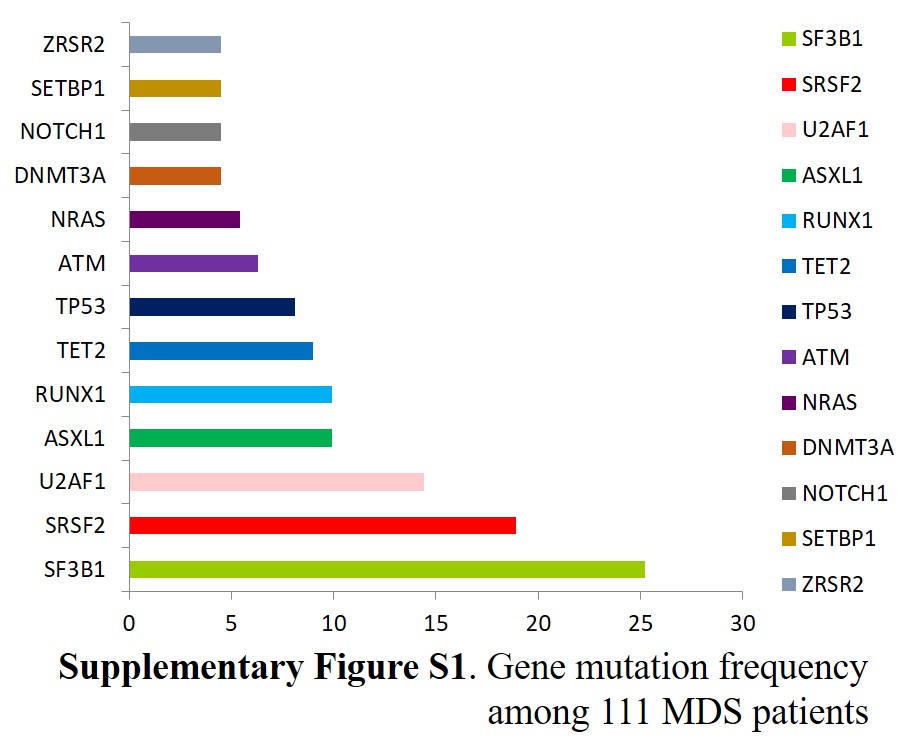

Supplement: Supplementary file 1 — Supplementary Information 1. [file 41598_2022_9864_MOESM1_ESM.jpg]

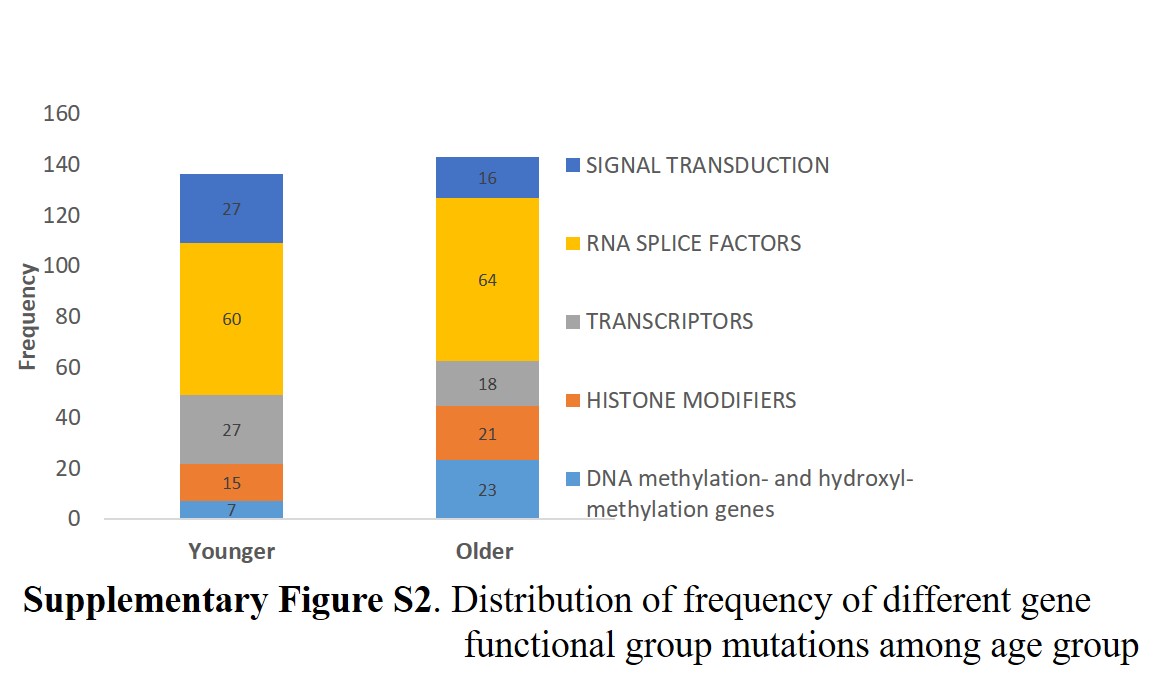

Supplement: Supplementary file 2 — Supplementary Information 2. [file 41598_2022_9864_MOESM2_ESM.jpg]

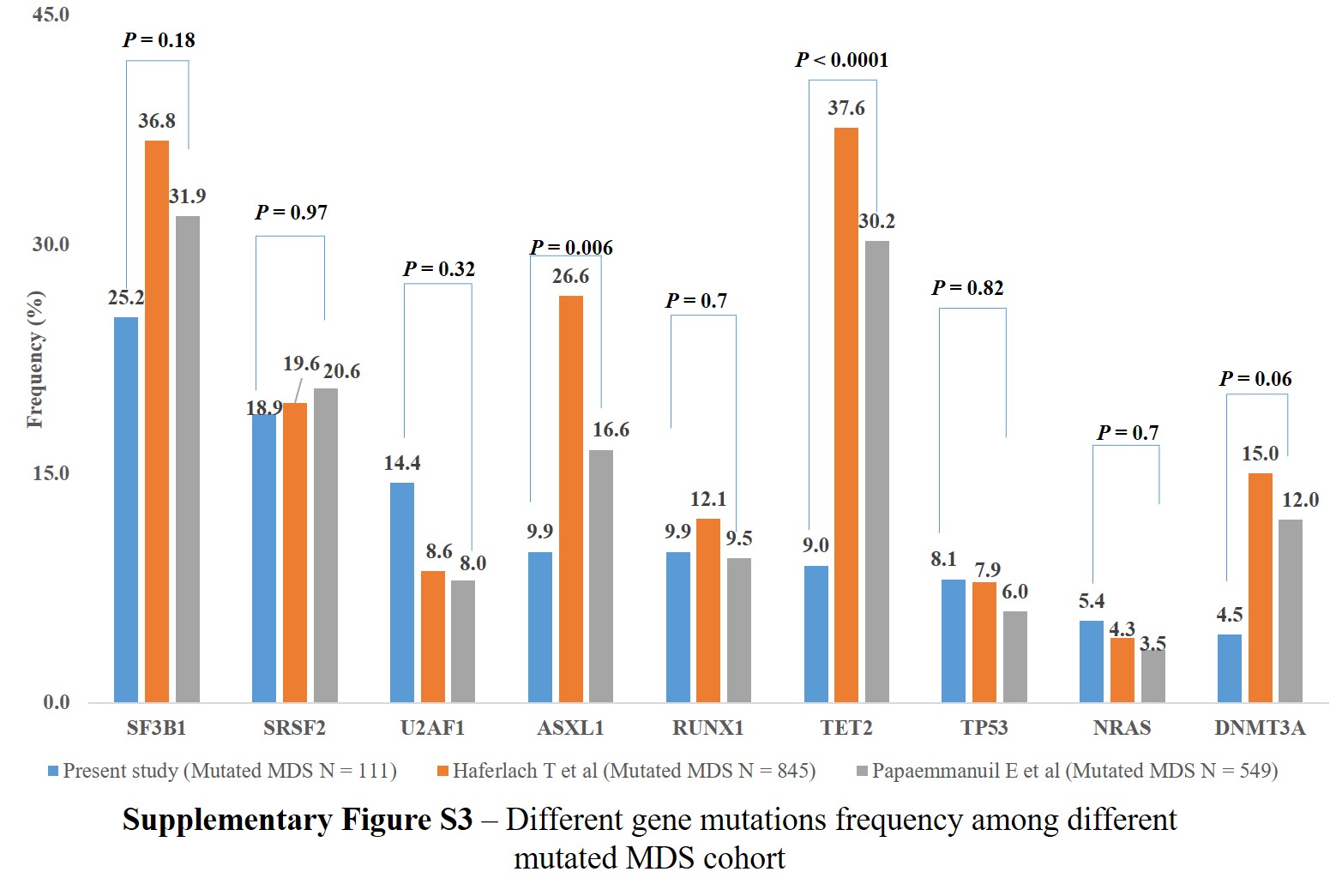

Supplement: Supplementary file 3 — Supplementary Information 3. [file 41598_2022_9864_MOESM3_ESM.jpg]
